# Multiple midline defects identified in a litter of golden retrievers following gestational administration of prednisone and doxycycline: a case series

**DOI:** 10.1186/s12917-018-1419-y

**Published:** 2018-03-12

**Authors:** Joanna L. Kaplan, Catherine T. Gunther-Harrington, Jessie S. Sutton, Joshua A. Stern

**Affiliations:** 1 WR Pritchard Veterinary Medical Teaching Hospital, School of Veterinary Medicine, University of California Davis, One Shields Ave, Davis, CA 95616 USA; 2 Department of Medicine & Epidemiology, School of Veterinary Medicine, University of California Davis, One Shields Ave, Davis, CA 95616 USA; 3 Department of Surgical and Radiological Sciences, School of Veterinary Medicine, University of California Davis, One Shields Ave, Davis, CA 95616 USA

**Keywords:** Teratogen, Peritoneopericardial diaphragmatic hernia, Congenital heart defect, Gastroschisis, PPDH

## Abstract

**Background:**

The teratogenic effects of immunomodulatory and certain antimicrobial therapies are described in small rodents and humans. While the described teratogenic effects in small rodents have been extrapolated to make conclusions about its use in the pregnant dam, teratogenic effects of prednisone and doxycycline have not yet been reported in the dog. Here we report and describe midline defects observed in a litter of golden retriever puppies exposed to mid-gestational immunosuppressive and antimicrobial therapy.

**Case presentation:**

Twenty-one days into gestation, the dam of a litter of eight golden retriever puppies was administered prednisone, doxycycline, and tramadol as treatment for immune-mediated polyarthritis. The individuals in the litter were subsequently diagnosed with a variety of midline defects and congenital cardiac defects. This case series describes the variety of identified defects and presents a descriptive account of complex congenital abnormalities that are likely secondary to teratogenic effects of one or more drugs administered during gestation. The available puppies, dam, and grand dam underwent thorough physical examination, complete echocardiogram, and where indicated, advanced imaging with various surgical corrections when possible.

Numerous midline congenital defects and congenital heart disease were identified in the puppies evaluated. Ultimately 5 of 8 puppies born to the dam were presented for thorough evaluation. The midline defects include: gastroschisis (1), peritoneopericardial diaphragmatic hernias (4, PPDH), umbilical hernia (4), unilateral cryptorchidism (1 of 4 males), cleft palate (1), renal agenesis (1), renal abnormalities (1), sternal and vertebral abnormalities (3), remnant liver lobe (1) and malformations consistent with ductal plate malformations with congenital hepatic fibrosis (1). The congenital cardiac defects include: ventricular septal defect (4, VSD) and subaortic stenosis (4, SAS). The presence of greater than one congenital defect was noted in all 5 of the dogs evaluated. Surgical correction was necessary for PPDH in 4 puppies. Medical intervention was recommended for congenital cardiac disease in 1 puppy.

**Conclusion:**

This case report is the first to describe midline defects in dogs that have been exposed to immunomodulatory therapy during gestation. A causative relationship between mid-gestational immunomodulatory exposure and midline defects cannot be proven, however, this case supports a clear association and provides case-based evidence to support its avoidance when possible.

## Background

A 6-year-old golden retriever primiparous dam gave birth to a litter of eight puppies with multiple midline defects (Table 1). The sire had no reported midline defects or cardiac abnormalities in multiple previous litters. At 3 weeks gestation, the dam developed suspect immune-mediated polyarthritis (IMPA) that resolved following empirical treatment. Treatment included doxycycline 4.7 mg/kg orally every 12 h for 30 days, tramadol 3.1 mg/kg orally every 12 h for an unknown duration, and an immunosuppressive dose of prednisone of 0.95 mg/kg orally every 12 h for 10 days tapered gradually over 20 days. The puppies were carried to term and delivered without complications. At the time of birth 8 puppies were delivered. One was born with a gastroschisis with a 1 cm abdominal wall defect and a cleft palate. This puppy was found deceased 12 h after birth. A second puppy was born with a decreased birth weight of 0.22 kg and was found deceased 24 h after birth. No necropsies were performed. At 15 weeks of age, 4 of the remaining 6 puppies were presented to the Cardiology Service at the University of California, Davis Veterinary Medical Teaching Hospital (UCD VMTH) for murmur evaluation (murmur was noted in 1 puppy at a routine exam). The dam and grandmother of the litter were evaluated during the same visit and determined to be clinically normal based on physical examination and complete echocardiogram evaluation. The following case report describes the clinical outcome in 5 of the puppies. This is the first case report to describe dogs born with multiple midline defects after mid-gestational exposure to prednisone, doxycycline and tramadol.

**Table 1:** Individual dogs are reported with an “X” denoting their respective congenital defects. For each congenital defect or defect category reported a total is provided. For each dog evaluated, a total number of defects is provided

	Abnormalities										
Dog	Gastroschisis or umbilical hernia	PPDH	VSD	SAS	Skeletal malformation	Liver malformation	Renal malformation	Decreased birth weight	Cleft palate	Unilateral cryptorchidism	Total number of abnormalities
1			X	X						X	3
2	X	X	X	X	X	X					6
3	X	X	X	X	X	X	X				7
4	X	X	X		X		X				5
5	X	X		X							3
6	X								X		2
7								X			1
8											Not evaluated
Dam											0
Granddam											0
Total	5	4	4	4	3	2	2	1	1	1	27

## Case presentation

### Dog 1

**Fig. 1:**
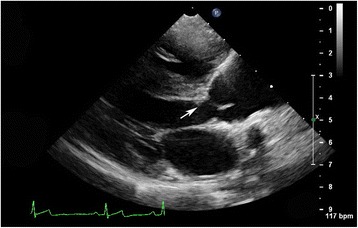
Echocardiographic image from the right parasternal long-axis view of Dog 1. There is a hyperechoic subaortic ridge present (denoted by arrow) as well as poststenotic dilation of the aorta consistent with subaortic stenosis (SAS). The left ventricular walls appear thickened consistent with concentric hypertrophy secondary to the pressure overload from SAS

At 7.5 weeks of age an incidental murmur was detected as was unilateral cryptorchidism during a routine wellness visit. At 8 weeks of age, he was presented to the Cardiology Service at the UCD VMTH for further evaluation. On physical examination, a grade I*V*/VI left basilar systolic ejection murmur, a grade IV/VI right parasternal systolic harsh murmur, and femoral *pulses parvus et tardus* were noted. An echocardiogram revealed severe SAS characterized by a hyperechoic subaortic ridge (Fig. 1) and left ventricular outflow tract velocity (LVOT Vmax) of 4.7 m/s. A restrictive, left to right shunting, perimembranous ventricular septal defect (VSD) was detected with a velocity of 4.5 m/s across the defect. No arrhythmias were appreciated on lead II electrocardiogram (ECG) during the echocardiogram. An agitated saline intravenous contrast study was performed and no evidence of a right to left shunt was noted. Atenolol was prescribed with dose increases to maintain a dose of 0.8–0.9 mg/kg q12 as the puppy grew.

Reevaluation at 15 weeks of age revealed progression in the murmur intensity, and atenolol was dose escalated to achieve a 1.67 mg/kg q12 dose. During reevaluation at 20 weeks of age, thoracic radiographs were performed which revealed marked aortic enlargement and no obvious skeletal malformations. Echocardiogram during this visit showed progressive left ventricular concentric hypertrophy and the LVOT Vmax remained severely elevated. The previously reported VSD now had a velocity of 3.8 m/s. Occasional single ventricular premature complexes (VPCs) were noted on ECG during the echocardiogram. Dog 1 remained subclinical at subsequent follow-up examinations. At 39 weeks of age echocardiogram revealed no new changes. Unilateral cryptorchidism persisted throughout evaluations. At the time of writing, dog 1 is 3 years old and is reported to have occasional syncopal events based on a phone conversation with the owner.

### Dog 2

Dog 2 was born with a 1 cm rent in the body wall at the umbilicus. The rent was surgically corrected immediately following parturition and revised due to dehiscence. Dog 2 reportedly underwent cardiac arrest while under general anesthesia during the second surgery and was successfully resuscitated. At 13 weeks of age, a grade III/VI left systolic murmur was noted in the record. A three-view thoracic radiographic study revealed generalized cardiomegaly largely characterized by right-sided enlargement. A complete blood count revealed a low hematocrit (HCT) of 32.3% (38.3–56.5%), low hemoglobin of 11.2 g/dL (13.4–20.7 g/dL), low red blood cells of 5.08 M/uL (5.39–8.70), and lymphocytosis of 7069/uL (1060–4950/uL). A chemistry panel revealed an elevated alkaline phosphatase (ALP) of 262 u/L (5–160 u/L), hypochloremia of 106 mmol/L (108–119 mmol/L), hyperphosphatemia of 9.7 mg/dL (2.5–6.1 mg/dL), hyponatremia of 141 mmol/L (142–152 mmol/L), hypoglobulinemia of 2.3 g/dL (2.4–4.0 g/dL) and elevated creatine kinase of 284 U/L (10–200 U/L). The values of the erythrogram, elevated ALP and phosphorus were considered normal given the puppy’s age. The lymphocytosis may represent an epinephrine-stress response or underlying chronic inflammation. Mild electrolyte abnormalities may be related to normal variation or the puppy’s lack of appetite.

Dog 2 was first evaluated by the UCD VMTH at 15 weeks of age. The owner reported a history of vomiting and mucoid diarrhea that waxed and waned with no history of exercise intolerance or decreased level of energy, appetite, or thirst. On physical examination, a grade I*V*/VI left basilar and right apical systolic heart murmur was detected. The puppy had a regular rhythm and adequate femoral pulse quality. An umbilical hernia was observed. No cleft palate or other obvious midline defects were noted. Echocardiogram was consistent with a PPDH. Liver and fat were noted within the pericardium. A small hemodynamically insignificant perimembranous VSD was visualized with a left to right shunt velocity of 3.0 m/s. The VSD was suspected to be restrictive, but the presence of abdominal contents within the pericardial sac hindered proper alignment with the shunt. A hyperechoic subvalvular ridge and mild left ventricular hypertrophy were identified. A LVOT Vmax of 2.8 m/s was noted, consistent with mild SAS.

At 19.5 weeks of age, dog 2 was presented to the Soft Tissue Surgery Service at UCD VMTH for surgical repair of the previously diagnosed PPDH and ovariohysterectomy (OVH). Preoperative bloodwork revealed a mild eosinophilia of 2210/ uL (70–1490/uL), lymphocytosis of 8840/uL (1060–4950/uL), elevated ALP of 271 u/L (5–160 u/L) and elevated alanine transferase (ALT) of 126 u/L (18–121 u/L). To evaluate for causes of eosinophilia and lack of a stress leukogram an ACTH stimulation test was performed. The results were inconsistent with hypoadrenocorticism (pre-cortisol 0.6 μg/dL, post cortisol 9.0 μg/dL). Repeated fecal examinations were also performed which were consistently negative. Thoracic radiographs revealed an enlarged cardiac silhouette with a dorsal peritoneopericardial mesothelial remnant (DPMR) and lack of the liver within the abdomen consistent with the diagnosis of PPDH. Additionally, radiographs revealed aortic dilation likely related to the diagnosis of SAS.

An exploratory laparotomy, corrective herniorrhaphy, and OVH were performed. The caudal sternebra was under-developed and bifurcated. The left medial and quadrate liver lobes were herniated through an approximately 3 cm defect in the diaphragm into the pericardial sac. Many adhesions were present. The spleen was irregular in shape with evidence of capsular fibrosis. The common bile duct was moderately dilated and the gallbladder could not be identified or located.

The liver lobes were gently reduced into the abdomen and the diaphragmatic defect was closed using a bilateral diaphragmatic rotational flap. An OVH was performed without complication. Dog 2 recovered from anesthesia uneventfully.

Due to persistent gastrointestinal signs a focal abdominal ultrasound was performed 2 weeks post-procedure and a gallbladder could not be identified. This was consistent with the intraoperative findings. Recheck echocardiogram at 39 weeks of age revealed static SAS and aneurysmal tissue in the area of the previously reported VSD effectively patching the defect.

By 2 years of age the dog began experiencing seizure episodes. Chemistry panel revealed an elevated ALT 393 (21–72), increased aspartate aminotransferase AST 70 (20–49), low blood urea nitrogen (BUN) 8 (11–33) and hypoproteinemia 5.3 (5.4–6.9). Serum bile acid assessment suggested hepatic dysfunction (pre and post values of 351.6 and 1.8 respectively). Serum ammonia level was increased [118 μg/dL (0–59)] and an abdominal ultrasound revealed evidence of portal hypertension and a small volume of peritoneal effusion. An extrahepatic or intrahepatic portosystemic shunt could not be identified on ultrasound. Three phase computed tomographic imaging of the abdomen revealed multiple acquired extrahepatic portosystemic shunts likely due to portal hypertension. A laparoscopic biopsy of the liver revealed moderate chronic multifocal lobular collapse with portal to portal bridging fibrosis, arteriolar reduplication, biliary hyperplasia, reduced portal vein profiles, and oval cell hyperplasia consistent with ductal plate malformation and congenital hepatic fibrosis. Acquired extrahepatic portosystemic shunts were therefore likely acquired secondary to fibrosis [1]. Medical management was initiated (amoxicillin 500 mg orally q12h, lactulose 4.5 mls orally q12h, and omeprazole 20 mg orally q24h). A 3-day course of tramadol 100 mg orally q8-12 h was initiated for sedation and pain management following laparoscopic biopsy. Echocardiogram during this visit revealed no significant changes.

At the time of writing, dog 2 occasionally develops ascites due to liver disease, which is therapeutically drained.

### Dog 3

**Fig. 2:**
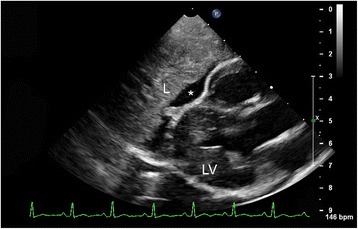
Right parasternal echocardiographic image of Dog 3. There is a small volume of hypoechoic pericardial effusion (denoted by asterisk) and soft tissue structure most consistent with liver (labeled as L) within the pericardial space, adjacent to the heart (left ventricle labeled as LV)

Evaluation was performed at 15 weeks of age. The puppy had a history of soft mucoid stool, several episodes of shallow panting while at rest, and was calmer than the other littermates. On physical examination, a 2 cm, non-reducible umbilical hernia was noted. No cardiac structural or functional abnormalities were identified on echocardiogram, however a PPDH was observed with liver identified within the pericardial sac that limited the cardiac examination (Fig. 2).

**Fig. 3:**
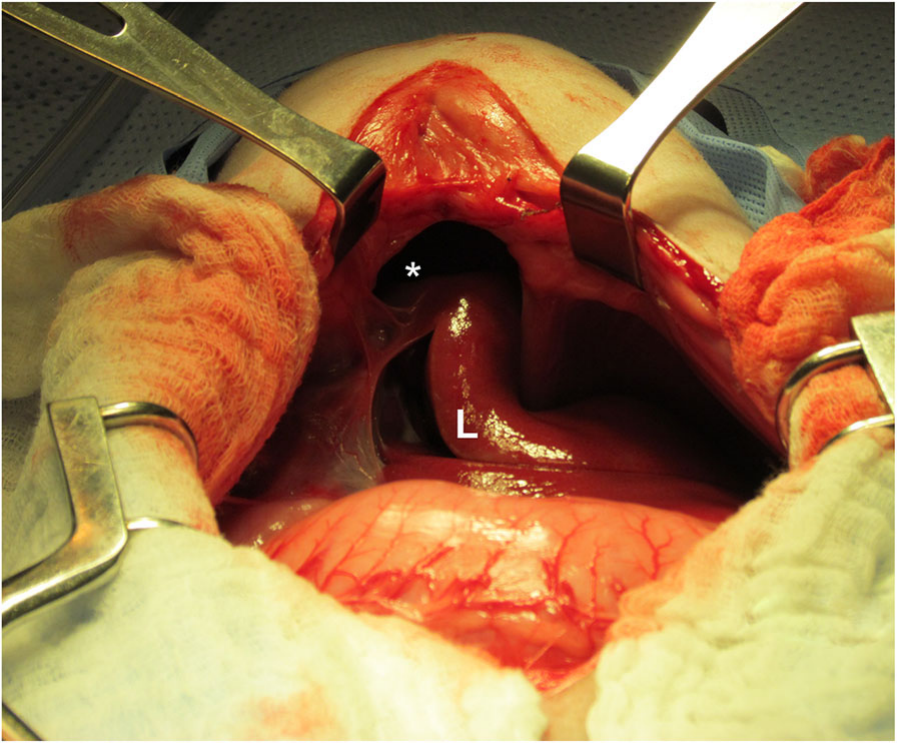
Image from the laparotomy performed on Dog 3. There is a 4x6cm defect through the central tendon of the diaphragm that communicates with the pericardial sac, consistent with a peritoneopericardial diaphragmatic hernia. A portion of the right medial liver lobe can be seen herniated into the pericardial sac

A CBC and chemistry panel performed at 19 weeks of age were unremarkable. A follow-up echocardiogram performed at this time revealed a small perimembranous VSD sealed by aneurysmal tissue and high normal aortic outflow velocity of 1.98 m/s raising concern for equivocal or mild SAS in a puppy of this age. Thoracic radiographs were consistent with a PPDH. Only 7 sternebrae segments were present. An exploratory laparotomy, herniorrhaphy, cranioventral abdominal wall defect repair, and castration were performed. Intra-operatively, the surgeon noted the appearance of the caudal sternebrae to be subjectively abnormal and xiphoid process remained cartilaginous and underdeveloped in appearance. A portion of the right medial liver lobe and gallbladder were herniated into the pericardial sac. The herniation was through an approximately 4–6 cm defect in the diaphragm into the pericardium (Fig. 3). The quadrate liver lobe was hypoplastic. Left renal agenesis was also noted, although the left adrenal was present. The herniated liver lobe and gallbladder were reduced manually into the abdomen and the defect was closed using a bilateral diaphragmatic rotational flap.

During a follow-up examination at 22 weeks of age, the owner reported that dog 3 was doing excellent at home with an improved level of energy and no vomiting or diarrhea. At the time of writing, dog 3 is alive and apparently healthy based on a phone conversation with the owner.

### Dog 4

During the initial evaluation at 15 weeks of age, Dog 4 was reported asymptomatic. On physical examination an umbilical hernia was present and gut sounds auscultated in the thorax. No heart murmur was noted. An echocardiogram was performed, revealing a PPDH consisting of dilated air-filled small intestinal loops within the pericardial sac. No functional or structural cardiac abnormalities were identified. However, cardiac evaluation was limited due to acoustic artifact from air present within the pericardial space.

**Fig. 4:**
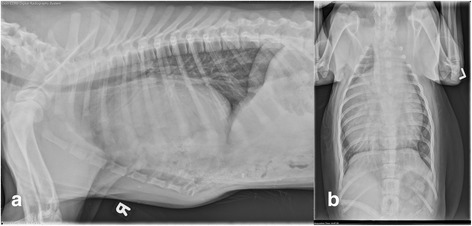
Right lateral (**a**) and dorsoventral (**b**) thoracic radiographs from Dog 4. The cardiac silhouette is markedly enlarged and rounded and there are visible intestinal loops containing granular and soft tissue opaque material superimposed on the ventral cardiac silhouette. There is border effacement of the ventral margin of the diaphragm, and the intestinal loops superimposed on the cardiac silhouette are confluent with the abdomen. These findings are consistent with a peritoneopericardial diaphragmatic hernia with suspect herniation of intestinal loops and potentially liver

At 19 weeks of age, pre-operative bloodwork was performed which revealed a mild leukocytosis of 18.2 K/uL (4.9–17.6 K/uL) characterized by a lymphocytosis of 8245/uL (1060–4950/uL), hyperphosphatemia 9.0 mg/dL (2.5–6.1 mg/dL), and a slightly elevated creatine kinase 208 U/L (10–200 U/L). Thoracic radiographs were consistent with a PPDH with suspect herniation of intestinal loops and liver (Fig. 4). A repeat echocardiogram performed the day before surgical repair revealed a small, restrictive, perimembranous VSD with left to right shunting and mild tricuspid valve regurgitation. An exploratory laparotomy, herniorrhaphy, umbilical hernia repair, and castration were performed. Upon exploratory laparotomy, there was an additional pinpoint 2 mm defect present in the cranial abdominal wall on midline near the umbilicus, and a 4 cm defect in the diaphragm communicating with the pericardial sac (Fig. 4). The xiphoid region was subjectively abnormal and the transversus thoracicus was noted to be more caudal than normal with midline asymmetry. The right-sided muscle was displaced caudal compared to the left. Abdominal contents found herniated into the pericardial sac included duodenum, the majority of the jejunum, half of the left lateral liver lobe, half of the left medial liver lobe, gallbladder, quadrate lobe, and a portion of the right medial liver lobe. The right kidney was observed to be smaller than the left kidney. An incidental cloth foreign body was palpated in the jejunum and consequentially removed by conventional simple enterotomy as previously described [2]. After abdominal organs were reduced back into the abdominal cavity, excess pericardium was excised and the pericardial sac was closed. The edges of the diaphragmatic defect were closed with bilateral diaphragmatic rotational flaps. The umbilical defect was repaired during closure of the abdominal incision. Castration was performed without complication.

Three weeks post-procedure (at 22 weeks of age) Dog 4 was evaluated for inappetence. On physical examination he was hyperthermic (rectal temperature 103.5 degrees Fahrenheit), tachypneic, and tachycardic. A chemistry panel revealed hypoglycemia 63 mg/dL (77–150 mg/dL) and 1.5 mls of 50% dextrose was administered orally. An automated CBC revealed a marked leukocytosis of 31.98 K/uL (5.05–16.76 K/uL) characterized by a neutrophilia of 27.83 K/uL (2.95011.64 K/uL) and a suspected left shift. Full body radiographs were suspicious for marked gastroenteritis. A parvovirus SNAP test was negative. Dog 4 was referred to the UCD VMTH for further care. A second parvovirus SNAP test and parvovirus PCR test were performed. Both were positive for parvovirus infection. The puppy was treated with aggressive supportive care and he recovered fully.

At the time of writing, dog 4 is alive and apparently healthy based on a phone conversation with the owner.

### Dog 5

Dog 5 was first evaluated by the UCD VMTH at 22 weeks of age. A physical examination revealed borborygmi that were auscultated in the thorax, a 1.5 cm umbilical hernia, and linguoverted mandibular canines. A complete blood count and chemistry panel were normal for a puppy [HCT of 37.5% (40–55), elevated phosphorus 7.8 (2.6–5.2), total calcium 11.8 (9.6–11.2), and ALP 187 (14–91)] with the exception of a mildly elevated ALT 81 (21–72). A urinalysis was unremarkable (USG 1.051 and pH of 6). An echocardiogram revealed a PPDH and mild SAS, but the heart was difficult to evaluate due to the presence of bowel loops within his pericardium. An exploratory laparotomy, herniorrhaphy, and castration were performed. There was a 3 cm defect in the ventral aspect of the muscular portion of the diaphragm, which communicated with the pericardium. The small intestine, cecum, colon, and liver were within the pericardial sac. These organs were reduced into the abdomen and the defect was closed in a simple continuous appositional fashion. Castration was performed without complication. Dog 5 recovered uneventfully from anesthesia. Prior to surgery, dog 5 had vomited up a pair of socks, but no other foreign objects were found during surgery. No recheck echocardiographic data is available.

At the time of writing, dog 5 is alive and apparently healthy according to owner during a phone conversation.

### Dam & Grand Dam

The dam and grand dam were evaluated by the UCD VMTH Cardiology Service during the litter’s initial visit. Both the dam and grand dam had normal heart structure and function on echocardiogram with an LVOT Vmax’s of 1.83 m/s and 1.87 m/s, respectively. Fractional shortening was 30 and 33%, respectively. No midline defects or cardiac shunts were identified.

The remaining live puppy of the litter was unavailable for evaluation by the UCD VMTH Cardiology Service.

## Discussion and conclusions

In this case series, the dam developed a presumed gestational IMPA, which prompted treatment with prednisone, doxycycline, and tramadol. The resulting litter of puppies developed multiple midline defects. The prescribed treatment and resultant defects appear correlated, however it is impossible to determine if these results are specifically due to the drug administration, the dam’s immune-mediated disease, hereditary effects, genetic susceptibility, epigenetics, or another unidentified cause. In addition because 3 drugs were given it cannot be determined if the midline defects were due to the prednisone, doxycycline, tramadol, or an effect of the three drugs administered concomitantly. The inability to control these multiple variables is one of the major limitations of this case series. However, ethical considerations make it impossible to design a prospective canine study in which these variables can be controlled. The null hypothesis that the litter of puppies developed congenital disease unrelated to any environmental factors must be considered. However, the number and variety of defects affecting all puppies is less typical of a genetic mutation and more characteristic of a teratogen.

The midline defects observed included a cleft palate, gastroschisis, umbilical hernias, a large abdominal hernia, ventricular septal defects, cryptorchidism, malformation of the xiphoid and transversus thoracicus, spina bifida, renal dysplasia, renal agenesis, hypoplastic liver lobe, malformations consistent with ductal plate malformations and congenital hepatic fibrosis, and PPDHs. Other congenital cardiac defects noted included SAS. A review by Evans and Biery described the history, clinical signs, radiographic and surgical findings in 13 dogs with PPDHs [3]. Concurrent umbilical hernias were noted in 4 dogs, congenital cardiac disease in 2 dogs, and sternal deformities in 6 dogs. Surgical correction was successful in 9 of the 11 dogs examined and clinical signs remained only in 2 dogs. The review reported that the organs most frequently herniated into the pericardial sac include the gallbladder and liver lobes. Small intestinal loops, stomach and spleen were less commonly herniated into the pericardial sac. Mesentery, omentum, and the falciform ligament were rarely observed in the pericardial sac. These findings were consistent with the defects identified in this case series. Unlike the current case series, there was no history of reported disease or drug administration in the dams during pregnancy.

Two of the dogs in this case series had sternal abnormalities which is commonly reported in dogs with PPDH. While, the time period of sternal ossification specifically in the dog has not been well-described, one report in the cat describes sternal ossification occurring on days 28–32 of gestation [4, 5]. At the time of PPDH correction surgery in one of the dogs, the xiphoid process remained cartilagenous, suggesting a failure of ossification.

Subaortic stenosis was identified in 4 of the dogs evaluated. SAS is overrepresented in the golden retriever and has been demonstrated to be familial, making up approximately 15% of cases [6]. Thus it should be considered that development of SAS in these puppies may be unrelated to the other defects. However, the concurrent finding of a VSD in all the puppies may suggest that at least a component of their congenital heart disease was indeed due to teratogenic exposure. While multiple congenital malformations have been reported with SAS, including VSDs, in previous studies, it is unknown if these dogs had additional midline defects unrelated to the heart as the clinical history and presentation of these dogs was not reported [6, 7]. It is interesting to note that the dam and grand dam did not have any evidence of subaortic stenosis. However, a cardiology examination was not performed on the sire.

It is important to recognize that the total tabulation of defects may be incomplete. In addition to having one dog without complete evaluation, one of the puppies did not have a peritoneopericardial diaphragmatic hernia and so an abdominal ultrasound and/or an exploratory laparotomy were not performed. There could have been other midline defects present in these 2 puppies that went unrecognized. The sire of the litter could not be evaluated. Therefore, it is unknown if any congenital defects detected in this litter were related to undetected hereditary diseases that may have been vertically transmitted from the sire, or current diseases or drug administration during conception. Although the sire was reported to produce multiple prior litters without any noted congenital defects, a full physical examination, echocardiogram, and abdominal ultrasound would be required to further characterize the health of the sire.

While a direct correlation cannot be made between the midline defects and the gestational administration of doxycycline, prednisone, and tramadol, the overwhelming number of defects in this litter should serve as a significant warning against at minimum the concomitant use of these medications during gestation. This is the first report in the dog of congenital defects associated with simultaneous gestational administration of prednisone, doxycycline, and tramadol. While it is generally recommended to avoid drug administration during pregnancy, there are certain circumstances in which their use must be considered [8]. The Food and Drug Administration (FDA) has made recommendations for human, canine, and feline drug administration based on the organization’s highest level of knowledge. Ideally, safety information about drug administration during pregnancy is based on randomized prospective studies in the specific species of interest. However, for ethical reasons, the reported safety of many drugs for human, feline, and canine medicine are often based on data extrapolated from laboratory rodents and mice rather than the direct species receiving the medication. This limits our level of confidence in the safety of medications we use during pregnancy and potential adverse effects on the fetus and dam.

In the current study, drug exposure occurred during the dam’s third week of gestation. This period of gestation is during the transition from stage 1 to stage 2 of pregnancy in which embryo implantation and fetal ossification occur [9]. Because this is a period of rapid fetal growth and development, exposure to exogenous drugs or chemicals at this time may cause skeletal, organ, limb, or neurologic abnormalities. In addition, the prednisone and the doxycycline were continued for a course of 30 days, resulting in this drug exposure from roughly week 3 to week 8 of total gestation time. The duration of tramadol administration was unknown. The most recent information reported regarding reproductive safety of doxycycline and prednisolone in the veterinary literature dates back to 1989 [10] and the teratogenic effects of doxycycline, prednisolone, and tramadol have not been reported in veterinary patients outside of the laboratory setting. Due to the paucity of information in the veterinary field, additional recommendations are based on the FDA’s guidelines in human medicine, rather than specific studies or case reports observed in the dog [9].

Tramadol is listed by the FDA as a pregnancy category C drug, indicating that while animal studies show adverse effects to the fetus, there were no studies that demonstrate these adverse effects in humans or specific animal species that the drug is labeled for. In laboratory animals, embryotoxicity and fetotoxicity was observed only at 3–15 times the recommended dose [11]. No teratogenic effects were observed at therapeutic doses or at doses that did not cause toxicity in the mother. A 2015 retrospective study in human medicine evaluated the outcome of offspring in pregnant women who received tramadol. There was weak positive correlation between the incidence of congenital cardiac defects in offspring and women who received tramadol while pregnant, exposing the teratogenic potential of tramadol. While causation cannot be concluded from this study, this finding indicates that we cannot rule out the potential for tramadol to cause teratogenic effects in mammalian species, including the dogs in this case series [12].

Doxycycline is listed by the FDA as a pregnancy category D drug, which indicates that there is evidence of fetal risk in animals and humans, and the drug is only indicated if needed for life-threatening or serious disease. According to a review article by Papich, doxycycline is defined as a class D drug in a separate categorical system, meaning its use is contraindicated in the pregnant dog because this drug has been shown to cause congenital malformations or embryotoxicity [8]. Tetracyclines have been associated with skeletal and teeth malformations, as well as teeth discoloration in the fetus [8]. While tetracyclines are considered teratogens, a study by Czeizel et al. looked specifically at doxycycline and its teratogenic potential [13, 14]. This case-control paired analysis revealed a statistically significant difference between the number of pregnant women treated with doxycycline who had infants with no birth defects [63 out of 32,804 (0.19%)] and number of pregnant women treated with doxycycline who had infants with birth defects [56 out of 18,515 (0.30%)]. However, this study did not show a statistically significant higher rate of congenital abnormalities with doxycycline treatment given during the second and third months of gestation.

Prednisone is reported as a class C drug for use in dogs and cats, meaning these drugs have potential risks and should only be used when the benefit of therapy clearly outweighs the risks [15]. Like tramadol, The FDA has also listed it a category C drug. More recently, corticosteroids have been changed to a category D drug in humans due to recent data suggesting that corticosteroids administered during the first trimester in animals and humans cause an increased risk for oral cleft palate formation, intrauterine growth restriction, and decreased birth weight [1, 14–17]. Board et al. describe a case in which a pregnant woman receiving a renal transplant with concurrent prednisone and azathioprine therapy had no evidence of teratogenic effects on the fetus [18]. Another case report described a pregnant woman with chronic liver disease that was treated with azathioprine and prednisone in whom no teratogenic effects were observed [19]. While these case reports do not support the teratogenic potential of prednisone, a report by Bongiovanni and McPadden observed that large doses of corticosteroid administration can lead to fetal resorption and decreased fetal size and viability [20]. Out of 260 pregnancies treated with cortisone or an analogue, they reported 8 stillborn infants, 1 abortion, and 15 premature infants; seven full-term infants were reported to show various disorders [20]. Only 2 infants in the study showed cleft palates and in both cases, steroids were administered prior to the fourteenth week of pregnancy at high doses. Similar data is observed in laboratory rodents. In one report pregnant mice treated with cortisone develop offspring with a cleft palate, with the highest incidence of cleft palates being between the tenth and eleventh days of gestation out of 19 days total [16, 17]. Notably teratogenicity of cortisone depended on the dose, time during gestation, and genetic strain of mice. Other defects reported include shortening of the head, mandible, spina bifida, and marked reduction in birth weight. It is interesting to note that the control group in this study had no incidence of cleft palates.

It is difficult to extrapolate data seen in laboratory animals because laboratory animal strains may have a higher number of steroid receptors compared to dogs and cats [15]. Nevertheless, the FDA advises to use prednisone during pregnancy with caution in all animal species. If anti-inflammatory or immunosuppressive therapy is necessary, prednisone is the preferred choice because it is relatively short-acting compared to longer acting corticosteroids, such as dexamethasone. This case series does not serve to change the guidelines for the use of prednisone in the pregnant dam, but to expose a knowledge gap in the safety data available for use of doxycycline and prednisone in the pregnant dog. In human medicine, pregnancy exposure registries are used to gather data about how prescription drugs affect pregnant women and their fetus. This model may serve useful in veterinary medicine to develop a stronger understanding of the teratogenic potential of prednisone, doxycycline, and tramadol. In the absence of extensive exposure registries for companion animals, case series such as this should serve as a clinical warning for the possible consequences of gestational drug use.

## Abbreviations

ALP: Alkaline phosphatase
ALT: Alanine transferase
AST: Aspartate aminotransferase
BUN: Blood urea nitrogen
CBC: Complete blood count
DPMR: Dorsal peritoneopericardial mesothelial remnant
FDA: Food and Drug Administration
HCT: Hematocrit
IMPA: Immune mediated polyarthritis
LVOT Vmax: Left ventricular outflow tract velocity
OVH: Ovariohysterectomy
PPDH: Peritoneopericardial diaphragmatic hernias
SAS: Subvalvular aortic stenosis
UCD VMTH: University of California Davis Veterinary Medical Teaching Hospital
USG: Urine specific gravity
VPCs: Ventricular premature complexes
VSD: Ventricular septal defect
